# Clients’ views and experiences of individual placement and support: an employment program for people in treatment for substance use

**DOI:** 10.3389/fpubh.2026.1892077

**Published:** 2026-08-05

**Authors:** Joanne Neale, Sarah Cosgrove, James Cassidy, Josh Dumbrell, Kevin Gardiner, Sophie McCluskey, Madeline Nightingale, Andrew Perkins, Samantha Steele, Kankan Zhang

**Affiliations:** 1 Addictions Department, Institute of Psychiatry, Psychology & Neuroscience, King’s College London, London, United Kingdom; 2 Centre for Social Research in Health, University of New South Wales Sydney, Sydney, NSW, Australia; 3 Figure 8 Consultancy Services Ltd, Dundee, United Kingdom; 4 RAND Europe, Cambridge, United Kingdom

**Keywords:** alcohol and other drugs, employment, employment program, implementation, Individual Placement and Support (IPS), qualitative, substance use, treatment

## Abstract

**Background:**

Individual Placement and Support (IPS) was originally developed to help people with serious mental illnesses into competitive employment but has recently shown positive results when delivered to people in treatment for substance use. This paper explores clients’ views and experiences of IPS for substance use to understand how the program is working across England, United Kingdom, and whether any improvements can be made.

**Methods:**

A longitudinal qualitative study with 48 new IPS clients was conducted between January 2024 and August 2025. Participants were interviewed up to four times each by telephone generating 153 interviews that were analysed inductively by Iterative Categorization. Findings were then mapped onto RE-AIM, an implementation science framework that assesses public health and community interventions.

**Findings:**

Participants’ accounts highlighted eight aspects of IPS (knowledge, engagement, outcomes, overall assessment, support received, valued components, future support desired, and potential improvements) which mapped onto four of the five RE-AIM dimensions (reach, effectiveness, implementation, maintenance). People who joined IPS for substance use tended to have limited knowledge and understanding of the program, but good engagement. They described multiple benefits, had few criticisms, and were overall positive. IPS provided them with practical employment-related assistance plus emotional support through strong relationships with IPS staff. Looking to the future, many participants wanted more of the same type of help from IPS, but also more in-work assistance and aid accessing education or training.

**Conclusion:**

Clients were very appreciative of IPS for substance use. Nonetheless, findings indicate that the program could be improved if more information was available to clients; measures of effectiveness included clients’ subjective views on their progress towards employment; additional resources were available to invest in IPS staff; and further in-work support was routinely offered (both for longer periods of time and to clients who exited substance use treatment).

## 1 Introduction

The relationship between substance use disorder (SUD) and employment is complex. People with SUD are more likely to become unemployed than people without SUD and people who become unemployed are more likely to develop a SUD than people who do not become unemployed (1–4). Unemployment and unstable work history are also risk factors for relapse after treatment for SUD (2, 4, 5). The reasons behind these associations are multifaceted and have been extensively researched. For example, people with SUD can encounter both individual and structural barriers to employment, such as having no or few qualifications, interrupted employment histories, poor health, insecure housing, stigma, and workplace discrimination (6–9). Meanwhile, substance use can negatively affect work performance and result in absenteeism leading to job loss (1, 9–11).

Despite these negative associations, there is evidence that people with SUD want to work and find working enjoyable (7, 8, 12). Additionally, securing a job and being employed has been shown to predict positive substance use and related treatment outcomes. Thus, people with SUD who are in work or who initiate work during treatment stay in treatment for longer, are more likely to complete treatment, have lower rates of relapse, and achieve higher rates of abstinence compared to unemployed clients (5, 7, 11). Reflecting this, research has suggested that employment can, *inter alia*, be a distraction from substance use and provide people with a way of filling their time constructively when substance use stops or decreases (8, 13).

Employment additionally has known beneficial effects on the daily lives and overall well-being of people in the general population and people who use substances (14, 15). For example, employment can offer financial rewards and economic stability, a sense of identity, social connections, and feelings of belonging (16–18). Being in work can also provide structure, routines, and balance in life; enable people to learn and develop new skills; and increase self-esteem, self-confidence, respect and pride (13, 17, 19). In contrast, unemployment is linked to poor physical and mental health and a range of other social problems (including family disruption, homelessness and crime) which are costly for individuals and for society (14, 17, 20, 21).

The benefits of employment notwithstanding, returning to and staying in work can seem daunting and overwhelming for people with SUD (15). Poor quality jobs and precarious employment can undermine health and wellbeing (22, 23) and some people with SUD worry that employment may undermine their recovery (20, 24). Being in work can make it difficult to attend treatment appointments; income from work may make procuring substances easier; and jobs in bars and restaurants can increase access to substances (6, 9). Furthermore, some workplaces have normalized cultures of substance use, and some jobs can be stressful or involve long hours, prompting declines in mental health and potentially increasing the risk of relapse (25–27). There is also historical evidence that people with SUD prefer a ‘recovery first/work second’ approach (28) and see full-time employment as a long-term, rather than a short-term, aim (8).

Responding to this complexity, various interventions have been developed to support people with SUD into work. They include workplace supported recovery programs, employment counselling programs, co-ordinated care and intensive case management, mutual help-oriented recovery homes, therapeutic workplaces, community restitution apprentice-focused training, drug court employment interventions, jobseekers’ workshops, integrated interpersonal cognitive problem solving, and Individual Placement and Support (IPS) (7, 20, 29, 30). These interventions generally target the individual rather than wider structural factors that undermine employment, such as limited job opportunities, stigma, or transport problems (15). Moreover, a systematic review completed in 2020 found that the effects of these interventions on employment tend to be small and warrant further study (7). IPS, including its variant Customized Employment Supports, is the intervention with the most empirical evidence supporting its implementation (7, 31).

IPS was originally developed to support people with serious mental illnesses (SMIs) in obtaining and maintaining employment in the open competitive labour market (16, 21, 32). It involves the provision of intensive, individually tailored assistance by an employment specialist to help people find their desired job. In-work support is then offered (to both employers and employees) to ensure that work is maintained (12). Although the evidence-base is still emerging and findings have sometimes been mixed, IPS has shown positive outcomes (including work attainment, longer time employed, and longer time employed in a single job) across a range of populations with SMIs (16, 33–36). Indeed, an overview of systematic reviews and meta-analyses of randomized controlled trials published in 2023 concluded that IPS is an evidence-based practice for people with SMIs in high income countries and shows potential in helping other populations (32).

A traditional IPS model follows eight principles, the combination of which contributes to its distinctiveness. Specifically, IPS aims to get people into competitive employment (access to education and training is not, however, a core component); it is open to all who want to work; it tries to find jobs consistent with people’s preferences; job search starts quickly; it brings employment specialists into clinical teams; employment specialists develop relationships with employers based on clients’ work preferences; it provides ongoing, individualized support for the person and their employer; and it includes counselling on welfare benefits (37). Participation in IPS does not *per se* affect welfare payments, but clients who start working or increase their hours may find that their benefits are affected, and so employment specialists routinely calculate income changes for clients. A 25-point fidelity scale is used to ensure delivery quality with higher scores denoting greater fidelity and increased expectation of job outcomes (38, 39).

When used in the context of alcohol and other drug treatment services in England, IPS is delivered with some minor modifications to both the principles and fidelity scale (40). Most notably, IPS clients need to be receiving structured treatment for their substance use, be of working age, and have the right to work in the United Kingdom. Additionally, instead of time-unlimited support, they are offered approximately 9 months of assistance with job-searching followed by 4 months of in-work support (although these are soft rather than hard limits). This timeframe was adopted based on findings from a trial that tested whether introducing a time limit for people receiving IPS for mental health problems altered its effectiveness (41). The results showed that the time-limited version was equally effective to the longer version and only minimal extra employment was gained when IPS lasted beyond 9 months (41).

IPS has now been successfully delivered to people in treatment for substance use in studies conducted in the United States (42) and Norway (43). In England, IPS was first implemented in substance use treatment services as part of a randomized controlled trial (the IPS-AD trial) that ran from 2018 to 2020 (21). The IPS-AD trial found that IPS helped more people secure employment within 18 months than standard employment support; although positive effects varied by area and benefits were not seen for those in treatment for opioid use disorder (21). Encouraged by these findings, the UK Drug Strategy committed to achieving full coverage of IPS in substance use treatment services across England by March 2025 (44). Administrative data have since provided further evidence for the effectiveness of IPS in alcohol and other drug treatment populations, including improved results for people in treatment for opioid use disorder (45).

Notwithstanding this literature, there is no peer-reviewed qualitative research focusing on how people in treatment for substance use view and experience IPS outside of a trial context. Aas et al. (16) conducted semi-structured interviews with 17 people who received IPS for substance use through participating in a randomized controlled trial in Norway, but their focus was on clients’ experiences, perceptions, and meanings of work rather than IPS *per se*. In contrast, various studies have explored clients’ views and experiences of IPS for people experiencing SMIs. This research has found that people with mental health problems tend to be positive about the program and the support they receive (46–49). In particular, they value the personalized and flexible approach of IPS (47, 50–52); the holistic (emotional and practical) support received (47, 48, 50, 51); and positive relationships with their employment specialists (47, 50, 51). Some, however, would have liked more support, including more follow-up once in work and better advice on welfare benefits (52, 53). This paper aims to address a current gap in understanding by exploring clients’ views and experiences of IPS for substance use to understand how the program is working and whether any improvements can be made.

## 2 Methods

Our data derive from longitudinal qualitative interviews conducted with IPS clients as part of a mixed methods evaluation of the implementation and delivery of IPS in community substance use services across England. Ethical approval for all components of the evaluation, including the client interviews, was secured from the RAND Corporation’s Human Subjects Protection Committee (HSPC) in November 2023. The wider evaluation (which comprised desk research, workshops, interviews, surveys, and case studies involving a range of stakeholders) has been published separately and confirmed that IPS was part of the treatment offer in almost all local authorities in England by 2025 (40). The evaluation also found that employment specialists were highly integrated within treatment services; for example, they often shared physical space, used the same case management systems, and were sometimes employed by the same organization as the treatment provider. Moreover, some employment specialists had a professional background in alcohol and other drug treatment (40). In this manuscript, we focus on, and provide a detailed analysis of, the client interviews.

Seven community substance use treatment services were identified as recruitment sites with input from policy leads at the Office for Health Improvement and Disparities (OHID) (a UK Government body). Site selection was designed to ensure diversity in terms of service location (different areas of England; rural and urban), length of time delivering IPS, and performance metrics. Staff at the seven services were asked to introduce the qualitative study to all new IPS clients (no exclusion criteria) and to give them a written study information sheet. Staff were also encouraged to support the recruitment of all genders and people from diverse backgrounds and with experiences of different substances to generate broad insights. The information sheet clarified that participation was voluntary, would not affect any support clients received from the service and would involve up to four telephone interviews (although they could withdraw from the study at any time). Telephone (rather than in-person) interviews were chosen to facilitate recruitment from multiple areas of England and to optimize study retention whilst minimizing research costs (since telephone interviews can be rearranged easily and cheaply).

The four interviews were scheduled to occur soon after starting IPS (Wave 1), 3 months after starting IPS (Wave 2), 6 months after starting IPS (Wave 3), and 12 months after starting IPS (Wave 4). If clients expressed interest and gave permission, their contact details were passed to the research team. Four team members, who themselves had lived experience of substance use and recovery and were also trained qualitative interviewers, then contacted all interested clients by telephone. They provided the information sheet again, explained the study in more detail, and clarified that they were independent of the IPS program. If clients were happy to proceed, the first interview was arranged. Team members employed different levels of self-disclosure of their own substance use whilst maintaining appropriate boundaries to avoid moving into a supportive role. To further build trust and rapport, the team member who first contacted the client conducted all their interviews and maintained contact with them between interviews.

Two team training sessions were organized and written interview briefing notes were shared before any interviews were conducted; fortnightly team meetings were held throughout the data collection period; and a fifth team member listened to all interview recordings and provided feedback to increase consistency of approach. In total, 48 IPS clients were recruited to the study, and 153 interviews were conducted between January 2024 and August 2025. These included 48 interviews (Wave 1); 39 interviews (Wave 2); 35 interviews (Wave 3); and 31 interviews (Wave 4). Together, this resulted in 102 h of interviewing. Several attempts were made to contact participants who did not respond to follow up text messages or phone calls, and, on occasions, team members also contacted the services through which participants had been recruited to ask them to pass on messages to contact the study team. A small number of participants rejoined the study through this approach.

The researchers who were conducting the interviews audio-recorded participants’ informed consent prior to each interview and then audio-recorded the interviews separately. Participants were given a study number (01–48) and offered £20 (as previously approved by the research ethics committee) to thank them after each interview they completed. All interviews were semi-structured and guided by topic guides that were used flexibly and adapted according to the Wave but covered the same broad topics. These were: participant’s background; substance use and treatment; views and experiences of employment; views and experiences of IPS; any life changes between interviews; and hopes and expectations for the future. All interviews were transcribed verbatim by a professional transcription service and analyses occurred in stages.

To begin, each interviewer listened to audio recordings of the interviews they had personally conducted and summarized the participants’ responses into pre-prepared Excel files. This was not intended to reduce the data into a quantifiable format. On the contrary, the interviewers used the Excel files to enter paragraphs of text outlining participants’ responses to the interview questions. There was one Excel file for each Wave of interviewing and separate sheets within each Excel file for sections of each topic guide. Every sheet had a row for each participant and columns for topics discussed within the topic guide. For example, the first Excel file (Wave 1) had an initial sheet (‘Participant’s background’) with columns including ‘Age’, ‘Area of current residence’, ‘Area born’, ‘Ethnicity’, ‘Sex’, ‘Relationship status’, ‘Children’ etc. The second sheet (‘Substance use & treatment’) then had columns including ‘Age of first substance use’, ‘First substances used’, ‘Additional substances used’, ‘First treatment’, ‘Current treatment’ etc.

Each Excel file was next analysed independently to generate cross sectional findings by Wave. To this end, three members of the research team analysed the columns of each sheet via a process of Iterative Categorization (54, 55). Iterative Categorization was chosen since it offers a transparent set of processes for analysing qualitative data, including large qualitative datasets, that heightens the credibility, reliability and trustworthiness of research findings. This is achieved by repeatedly cycling through the data, prioritizing participants’ accounts when creating codes and categories and generating a clear audit trail from raw data through to publication. A further benefit of Iterative Categorization is that it can be used when undertaking inductive analytical approaches (allowing themes to emerge organically from the data) and deductive analytical approaches (allowing themes to be mapped against pre-existing theories or frameworks) (54, 55).

In line with Iterative Categorization, data in all cells of each column were summarized into bullet points that were annotated with the participant’s study number and Wave number so that it was always possible to track who had made which point at which interview. Bullet points for each column were then grouped and regrouped iteratively so that themes emerged inductively from the data. When data were unclear or missing, the researchers went back to the original transcriptions and added any further information. Findings for each column were then summarized in one or two paragraphs of text. Once all columns in the Excel file had been analysed, the researchers merged the summaries together into one report for each Wave. The four Wave reports were next reviewed and approved by all team members for accuracy.

Following this, one researcher, with support from other team members, reviewed all four reports and extracted all text relating to participants’ views and experiences of IPS. The text extracts were merged into one Word document and colour-coded by Wave to make it easier to identify similarities and differences over time. At this point, all data were systematically reviewed again via Iterative Categorization, and this time eight recurrent topics relating to clients’ views and experiences of IPS were identified. These were (i) Knowledge about IPS; (ii) Engagement with IPS; (iii) IPS Outcomes; (iv) Overall assessment of IPS; (v) Support received from IPS; (vi) Valued components of IPS; (vii) Future support desired from IPS; and (viii) Potential improvements to IPS. Findings relating to each topic were written up, verbatim comments from the original transcriptions were added to highlight key points, and all researchers checked and approved the final account produced.

In the final stage of the analysis, the eight topics emerging from the analyses were mapped deductively onto the RE-AIM framework (56). RE-AIM is a widely used implementation science model that assesses public health and community interventions based on five broad dimensions. These are: reach (proportion of the target population that participate in an intervention), effectiveness (success rate if implemented as in guidelines; defined as positive outcomes minus negative outcomes), adoption (proportion of settings, practices, and plans that will adopt the intervention), implementation (extent to which the intervention is implemented as intended in the real world), and maintenance (extent to which a program is sustained over time). Traditionally, RE-AIM has been deployed as a quantitative tool to assess individual and organizational level factors affecting program impact. However, it can also be used in qualitative studies with program participants, such as ours, to better understand how and why programs have (or do not have) impact and to provide greater clarity on where improvements might be needed (57).

### 2.1 Participants

Demographic and substance use characteristics of the 48 participants were relatively stable across all four Waves, suggesting low attrition bias (see Table 1). At Wave 1, approximately one-third (35.4%) identified as female, increasing slightly at subsequent Waves to 41.0% (Wave 2), 40.0% (Wave 3) and 41.9% (Wave 4). Participants’ mean age was 39 years (Wave 1); 35 years (Wave 2); 39 years (Wave 3) and 38 years (Wave 4). Nearly all participants said they were White British (all Waves) and just over half stated that their main problem substance was alcohol (all Waves). There is insufficient published information on IPS clients to assess the representativeness of the 48 people recruited against the wider IPS population. However, national data indicate that treatment populations are mostly male, mostly aged 30 to 49 years, mostly white British, and more commonly in treatment for alcohol than other drugs (58).

**Table 1:** Participants’ self-reported demographic and substance use characteristics.

Characteristic	Wave 1 (soon after starting to work with IPS) *N* = 48	Wave 2 (3 months after starting to work with IPS) *N* = 39	Wave 3 (6 months after starting to work with IPS) *N* = 35	Wave 4 (12 months after starting to work with IPS) *N* = 31
Sex				
*Male*	31 (64.6%)	23 (59.0%)	21 (60.0%)	18 (58.1%)
*Female*	17 (35.4%)	16 (41.0%)	14 (40.0%)	13 (41.9%)
Age at recruitment in years				
*Mean (range)*	39 (20–64)	35 (20–64)	39 (20–64)	38 (20–64)
Ethnicity				
*White British*	42 (87.5%)	34 (87.2%)	31 (88.6%)	28 (90.3%)
*White Other*	3 (6.3%)	3 (7.7%)	2 (5.7%)	2 (6.5%)
*Black British*	2 (4.2%)	1 (2.6%)	1 (2.9%)	1 (3.2%)
*Multiracial*	1 (2.1%)	1 (2.6%)	1 (2.9%)	0 (0.0%)
Main problem substance				
*Alcohol*	25 (52.1%)	20 (51.3%)	19 (54.3%)	17 (54.8%)
*Opioids*	7 (14.6%)	7 (17.9%)	5 (14.3%)	5 (16.1%)
*Cannabis*	5 (10.4%)	4 (10.3%)	4 (11.4%)	4 (12.9%)
*Cocaine*	3 (6.3%)	1 (2.6%)	1 (2.9%)	0 (0.0%)
*Crack cocaine*	1 (2.1%)	0 (0.0%)	0 (0.0%)	0 (0.0%)
*Ketamine*	1 (2.1%)	1 (2.6%)	0 (0.0%)	0 (0.0%)
≥ 2 *substances*	6 (12.5%)	6 (15.4%)	6 (17.1%)	5 (16.1%)

At recruitment to the study, most participants reported that they had qualifications or had previously completed courses or training, and almost all said that they had worked at some point in the past. Nineteen had worked within the last 12 months; six had worked within the last 3 years; four had worked within the last 5 years; and two had worked within the last 10 years. Four had not worked for more than 10 years and four had never worked. Not all felt that their substance use had affected their employment histories; however, participants frequently identified other barriers to working, including - but not limited to - poor mental or physical health, criminal justice involvement, and insufficient qualifications. Again, there is no published information on the education and work backgrounds of IPS clients nationally to use for comparison purposes.

## 3 Findings

Findings were successfully mapped against four of the five RE-AIM dimensions: Reach, Effectiveness, Implementation, and Maintenance (see Table 2). The fifth dimension (Adoption) would normally require staff or service level data as it covers intervention uptake by services and professionals. Accordingly, Adoption was not included in our analyses.

**Table 2:** Study findings mapped against the RE-AIM dimensions.

RE-AIM Dimension	Study findings
Reach	Knowledge about IPS
	Engagement with IPS
Effectiveness	IPS outcomes
	Overall assessment of IPS
Adoption	Not covered (would require staff or service-level data)
Implementation	Support received from IPS
	Valued components of IPS
Maintenance	Future support desired from IPS
	Potential improvements to IPS

### 3.1 Reach

The Reach dimension of the RE-AIM framework focuses on participation of the target population in the intervention. Two themes from our data (‘Knowledge about IPS’ and ‘Engagement with IPS’) related to awareness and uptake of IPS and therefore sit under this heading.

#### 3.1.1 Knowledge about IPS

At Wave 1, participants tended to report relatively limited knowledge about IPS. Most stated that they had only heard about the program recently and this had not always been through their substance use treatment service. For example, some had discovered IPS through other services, their own research, or personal social networks. None reported receiving or reading any leaflets or other written or digital information. Participants were also often uncertain about the remit of IPS beyond the fact that it was designed to help people with alcohol and other drug problems gain employment. Whilst a few understood that the support offered was personalized for each client, others were unsure how IPS could help them. Indeed, two participants confused it with a housing service, and another did not understand the connection between IPS and their substance use. Reflecting this, participants often expected to learn more about IPS over time:

“[The employment specialist] just asked me a lot of questions about my life… about [my] drug use, about [my] mental health, what goals I want to achieve and what sort of work I want to do. So, I could tell you more after the next appointment. I can’t really tell you much about this one.” (Participant 02, Male, Wave 1)

By their second interview participants had better knowledge about IPS, although a few were still uncertain. When asked directly, most knew that it provided support for people with substance use related issues to gain employment. Nonetheless, one commented that she had not ‘really had a chance to look into it’, whilst others reported that they were unsure about how it worked, or the range of services offered beyond helping people with their CVs and preparing for interviews. Another participant said that she had not been aware that the focus of IPS was only on employment and now realised that the program did not meet her needs as her current priority was abstaining from alcohol. Additionally, participants sometimes struggled to name their employment specialist and/or confused them with their drug worker or occasionally with the researcher from the study.

By Wave 3, participants clearly understood that IPS was intended to support people with a history of substance use in seeking employment. However, many said that they were basing their understanding of what IPS was on the support they had personally received so far, rather than on what they had been told:

“[My understanding is] only from the things that I’ve been helped out with really, like with getting my CV updated, being helped to access the Indeed job site, and stuff like that. And obviously access to the carpentry course and obviously they helped me get in with the scaffolding.” (Participant 18, Male, Wave 3)

Participants who remained in the study and continued their engagement with IPS until Wave 4 articulated a more detailed understanding of IPS. Overall, they recognised that IPS was a personalized service that helped people who use substances to secure paid employment, and that employment specialists were able to communicate with employers on behalf of their clients. Occasionally, participants interviewed at Wave 4 added that IPS could help people access training courses, improve their CVs, practise their interviewing skills, and bolster their confidence. Indeed, one female recognised that people who had problems with alcohol and other drugs were likely to benefit from this extra support because they had often not worked for a long time:

“A lot of… people in addiction probably haven’t worked for years and along comes [employment specialist]… ‘Don’t worry, we’ll help you sort everything out.’ She [employment specialist] fully supports people. It’s fabulous, because without services like that, people would be lost.” (Participant 29, Female, Wave 4)

#### 3.1.2 Engagement with IPS

At the time of their first interview, many participants had already had two appointments with their employment specialist, and some had established ‘weekly’, ‘bi-weekly’, or ‘regular’ contact. Despite this, several participants had only met or spoken to their employment specialist once. By Wave 2 most participants were still engaged with IPS, and those who had disengaged primarily attributed this to their poor physical or mental health or wanting to focus on addressing their substance use or recovery prior to working. A few participants also stated that they were engaging with IPS less at Wave 2 because they were now in work or had paused their IPS engagement but were planning to re-engage once their health had improved:

“She [employment specialist] has put me on hold for twenty days, just to see what is going on with my back… I do want to find work, but I don’t want to rush into getting into work and then struggling on a daily basis with my back. But [employment specialist] were really understanding about it and she just said, ‘Look, you’re not doing anything wrong by not seeing me’ and stuff. She said, ‘Your health is the main concern’.” (Participant 30, Male, Wave 2)

At Wave 3, just under half the participants interviewed reported that they were still actively engaged with IPS. Meanwhile, a few others stated that they had not been in contact with IPS recently but were still officially on the program and had not been formally discharged. Reasons for disengaging varied and, again, were not always negative. Some said that they no longer required support because they had acquired the skills they needed or had secured a paid job that they liked. Others explained that ongoing substance use, mental health problems, or moving to a new area had contributed to them disengaging from IPS, or they had become ineligible for the service as they were no longer in treatment for their substance use:

“You’ve got to have a drink problem or a drug problem… I would go back and access [IPS] if that was possible, but it’s not unless I’m on drink and drugs and I don’t want to be on drink or drugs.” (Participant 04, Female, Wave 3)

Only a few participants were still actively engaged with IPS at their fourth interview. Participants who were not engaged confirmed that they had stopped working with the program because they had already secured paid employment, were not currently looking for work, or did not want support. Additionally, one participant explained that she had never formally disengaged from IPS but had not heard from her IPS worker for over a month, whilst another said that he was ‘taking a break’ as he had lost his motivation to search for jobs after a series of rejected applications. Participants who had disengaged from IPS by Wave 4 were, however, sometimes considering re-engaging. For example, a few who were not currently looking for work said that they would want support from IPS when they began their job search again. Meanwhile, two who were already employed were interested in re-engaging with IPS to look for a different job. This included one participant who wanted longer-term support from her employment specialist to help her return to nursing:

“Obviously I did ask… could I come back to her [employment specialist] in two years’ time to help with getting [back] to my nursing? Because obviously I don’t have a clue… She said, ‘Obviously once you’ve got a job, that’s it’, they have no more involvement with you, which obviously that’s a shame.” (Participant 43, Female, Wave 4)

### 3.2 Effectiveness

The Effectiveness dimension of RE-AIM considers how the benefits of the intervention outweigh any negative effects when the intervention is delivered faithfully. Our findings relating to ‘IPS Outcomes’ and ‘Overall assessment of IPS’ both provide insights into the benefits of IPS from the perspective of program participants.

#### 3.2.1 IPS outcomes

At the time of their first interview, participants tended to be focused on updating their CVs, looking for potential jobs, setting up and/or updating online accounts, or learning new skills. A few had already started to apply for work, attended interviews, or signed up to an employment agency. One was in contact with a potential employer, one had begun volunteering, and others were already on, or were about to start, relevant courses. One participant had found and accepted a job since engaging with IPS but said that he had achieved this independently of the program. Nonetheless, he still felt that IPS had given him the confidence he had needed to apply and then to accept the role. Other participants emphasized that engaging with IPS had increased their self-esteem and confidence or given them the motivation they required to keep job searching:

“I just feel more confident about my future… I can’t pinpoint one specific thing. I think it’s everything that she [employment specialist] has been telling me. They know what they’re doing, and they know what it takes to get somebody into full-time employment.” (Participant 24, Male, Wave 1)

By Wave 2, three participants had secured full-time work and five had secured part-time work. Twelve had also undertaken some form of education or training since their last interview. Most participants stated that their employment specialists had been instrumental in them securing their new job by keeping them informed about local employment opportunities, helping them to distribute their CVs, passing their details on to employers, and/or being generally supportive. Meanwhile, a few participants stated that they had found their job online independently of IPS but then discussed it with their employment specialists who had supported them with their application. When asked to describe how IPS had helped them, participants again emphasized that the program had increased their confidence, positivity and motivation for work.

Three months later, at Wave 3, six participants had secured full-time work and seven had secured part-time work. Eleven had also undertaken some form of formal education or training since their last interview. Whilst those in work stated that their employment specialists had helped them to secure work by providing practical support, some also reflected on how they had benefited from having open conversations about their interests and possible types of work that might suit them. Indeed, several participants emphasized that the less tangible emotional help that IPS had offered them was a key part of the ‘package’ that had increased their self-esteem and confidence so enabling them to secure a job:

“It’s just the support they give you, from start to finish… The whole package was really good, what they did for me.” (Participant 24, Male, Wave 3)

By their fourth interview, five participants had secured full-time work and six had secured part-time work. Eleven had also undertaken some form of education or training since their last interview. Again, many participants said that their employment specialist had helped them to secure their current job, whilst others stated that they had found their employment independently. For example, one participant had been told about his current job by his friends, another had found his job online and decided to apply because he already knew the employer, and a third had started volunteering for a charity and was then encouraged to apply for his current position by his colleagues. In addition, two participants had discovered their current jobs through their substance use treatment service. Whilst a few participants said that they had permanent contracts, others did not know how long they would remain in their current role or were anticipating a move within the next two years. Participants were, however, overwhelmingly positive about their current jobs, and repeatedly referred to the financial, psychological and social benefits of working:

“All the people I work with are lovely, dead friendly, and it’s a different environment for us to work in.” (Participant 12, Female, Wave 4)

#### 3.2.2 Overall assessment of IPS

From their very first interview, participants were mostly very pleased with the support they had received from IPS; often describing the program as ‘great’, ‘really helpful’, or ‘lifesaving’. Some participants stated that it was too early to make a fair assessment as they had only recently begun engaging with the program. Meanwhile, a few were uncertain about how much IPS would be able to help them, with just one participant expressing explicitly negative views. This participant had hoped his employment specialist would communicate with potential employers on his behalf, but the latter had so far only sent him job advertisements.

At Wave 2, participants continued to be overwhelmingly positive about IPS and expressed confidence that the program would help them find a job. Indeed, many commented that IPS had already been very helpful, they were grateful for the support received, and they could not find fault with their employment specialist. Several participants reflected on how far they felt they had already progressed in their goal of securing work and others reported a high level of optimism about the future. Accordingly, some said they were feeling ‘positive’ or ‘happier’ or had ‘more energy’, and one male contrasted his positive experience of IPS with his disappointment in the Jobcentre and another job seeking organization with which he worked:

“[Name of job seeking organization] don’t really do a lot in all fairness… They’ve not put me onto any particular job at all and neither have the Jobcentre actually.” (Participant 13, Male, Wave 2)

Enthusiasm for IPS remained high at Wave 3, with participants often describing the program as ‘amazing’ or ‘brilliant’. Nonetheless, a few participants felt that they had not benefitted as much as they might have done because they had not fully engaged and one person felt that his last meeting had been a ‘waste of time’ as his employment specialist had simply repeated the job searches he had already undertaken himself.

By their final Wave 4 interviews, most participants were still very positive about, and expressed great appreciation for, the support they had received from IPS. Indeed, one participant stated that engaging with IPS was ‘the best thing’ she had ever done. Despite these very favourable assessments, a few participants identified areas where they felt disappointed in IPS. For example, one participant was frustrated that he had been discharged from the program after deciding to look for training instead of a work role; another was disheartened after being told that she could not access IPS again in the future as she was now in employment; and a third felt that she would have benefitted from more follow-up sessions with her employment specialist after securing her job:

“She [employment specialist] did always say, ‘Oh I’m there if you need anything’… They [IPS] obviously helped initially, yeah, but then… yeah. Obviously, it just kind of dropped out.” (Participant 48, Female, Wave 4)

### 3.3 Implementation

The Implementation dimension of RE-AIM focuses on whether the intervention is delivered as intended within the real world. Our findings on ‘Support received from IPS’ and ‘Valued components of IPS’ both relate to how IPS was provided and experienced in practice.

#### 3.3.1 Support received from IPS

At Wave 1, many participants said that they were already receiving help creating, editing, or sending out their CVs. They had also often been supported in setting up and navigating job search websites, identifying potential jobs and employers, writing cover letters, and preparing for interviews. Some stated that they had received support in applying for courses or volunteering roles, whilst others commented that their employment specialist had provided them with employment-related information and discussed employment options with them.

After 3 months (at Wave 2), participants tended to report that initial meetings with their employment specialist had focused on discussing and identifying the type of work that they wanted. In subsequent meetings, employment specialists updated participants on relevant local job opportunities, helped them with their CVs, and assisted with job applications. A few participants added that their employment specialist had enabled them to enrol in educational courses or training or had provided them with broader support, including purchasing a laptop, setting up a bank account, or securing a driver’s licence or identification. Additionally, employment specialists had sometimes distributed CVs to potential employers, submitted job applications on participants’ behalf, or accompanied them to organizations or businesses so that they could enquire if there was any work available:

“She [employment specialist] offered to contact the manager… She said that she would come with me, or she could go and speak to him first, discuss her role and discuss what she thinks I’m capable of and why I would be good for that job and stuff.” (Participant 25, Female, Wave 2)

Participants who were still engaged with IPS at Wave 3 often said that interactions with their employment specialists had become more informal and personalized over time. Nonetheless, meetings still involved discussions about progress in searching for work and participants continued to receive help with job searching, CV editing, and/or completing applications. Employment specialists also provided on-going practical assistance such as accompanying participants to job interviews or helping them acquire work clothes. Furthermore, a few participants had received help accessing education or training or support with organizing their personal finances.

At Wave 4, participants reported that their employment specialists continued to provide them with a wide range of employment-related assistance. This included help in preparing their CVs, exploring suitable types of work, conducting job searches, completing applications, organizing the financial logistics of working (e.g., performing welfare benefit calculations or setting up bank accounts), and securing work-related certificates. A few also said that their employment specialist had helped them overcome transport problems by facilitating access to a bus pass or opportunities to learn how to drive. Furthermore, some participants explained how their employment specialist had helped them to feel more motivated to work and given them the confidence they needed to feel that employment was viable for them:

“They [employment specialist] helped me get a job and have the confidence to get a job… I know I can hold down a job… [But] it wouldn’t have been too long ago [that] I wouldn’t have thought that I could get up at half five in the morning and then go to bed at eleven at night having worked most of the day, and then still feel really buzzing from enjoying myself and doing a good job and things.” (Participant 17, Male, Wave 4)

#### 3.3.2 Valued components of IPS

From their very first interviews, participants almost universally recognised the value of their employment specialists, whom they said made them feel more confident about themselves and their work prospects, as well as happier, more hopeful, and more optimistic about the future. In addition, some participants were grateful that their employment specialists encouraged them to think about jobs they had not previously considered, gave them a structure for the day, and took the time to get to know them and their employment goals:

“I went to my first [IPS] appointment [and] I thought, ‘Yeah, maybe they might be able to do something.’ Because you’re not just in and out in two minutes, they get to know you a bit better and get to know your needs or what you are like as a person.” (Participant 39, Male, Wave 1)

By Wave 2, participants’ comments pointed to two valued components of IPS. First, they appreciated the practical support that the program afforded them. This included help with preparing CVs and completing job applications, receiving updates on relevant local job opportunities, and liaising with potential employers etc. Second, they emphasized the relationships and regular contact they had with their employment specialists. Illustrating this, one participant stated that his employment specialist always went ‘above and beyond’ to help him; another described her employment specialist as a positive role model in her life; and a third stressed how easy it was to talk to her employment specialist. Others reported that their employment specialists gave them emotional reassurance plus confidence and self-belief.

Participants continued to value both the practical employment-related support provided by IPS and the relationships they had with their employment specialists at Wave 3. In respect of the former, they were grateful for the range of support they had received with employment-related issues. In respect of the latter, many said that their employment specialists were ‘great’, ‘brilliant’, or ‘professional’ and often went ‘beyond their role’. For many participants, having an open and trusting relationship with their employment specialist was particularly important:

“She’s brilliant. I know I can be honest with her, talk to her, and she’s honest with me.” (Participant 10, Female, Wave 3)

At Wave 4, participants again expressed appreciation for both the practical support they had received from IPS and the relationships they had with their employment specialists who were routinely described as being ‘supportive’, ‘really helpful’, or ‘absolutely amazing’. One participant explained how he preferred the assistance he had received from IPS over that provided by other employment services because IPS was more personalized. Meanwhile, another, who had been struggling with poor mental health, said that he was particularly grateful that his employment specialist had taken the time to understand his needs and to signpost him to companies that were supportive of people with histories of both addiction and mental health problems:

“I did go for a couple of interviews with employers that accept people with addictions and people who struggle with their mental health, struggle with their stress… I mean, there are employers out there now that [are supportive]… It’s a lot better than what it used to be.” (Participant 03, Male, Wave 4)

### 3.4 Maintenance

The Maintenance dimension of RE-AIM addresses the extent to which a program is sustained over time. Our findings on both ‘Future support desired from IPS’ and ‘Potential improvements to IPS’ speak to the likely continuation of IPS over the longer-term.

#### 3.4.1 Future support desired from IPS

At the end of their first interview (Wave 1), participants were asked about the type of support they would like from IPS in the future. Most said that they wanted weekly in-person appointments with their employment specialists, although some thought fortnightly or monthly meetings would be better. One person added that she was happy to receive support by telephone whilst another said she wanted to be able to text her employment specialist between meetings. The main types of future assistance identified at Wave 1 related to preparing for employment; searching for work; and on-going support once they had secured a job:

“A: help me find something [a job] and structure my application; B: maybe tailor my CV so that it isn’t so broad spectrum because I’ve got so much experience in different areas in different fields… Then C: once I start [working], to offer me that support *in situ*.” (Participant 20, Male, Wave 1)

When asked the same question at Wave 2, participants often emphasized that they would like the help they were currently receiving to continue. Expanding upon this, participants made comments such as ‘I could do with all the help I can get’ or ‘I cannot do it on my own’. Others referred to tasks with which they particularly wanted help. These include assistance editing their CVs, preparing for interviews, renewing certificates for operating machinery, and accessing additional training courses. One participant also flagged that he would need ongoing support if he started working as he had not had a job for a long time:

“Ongoing support, after I potentially start in a local job, if I need it, which they have said is available… Yes, just in case I need help because I haven’t worked formally for anyone for a long time.” (Participant 01, Male, Wave 2)

Most participants who had not found employment by Wave 3 said that they wanted to continue receiving support from IPS for the foreseeable future. A few stated that they wanted someone who would stay in touch with them and who would be there to rely on if they ever needed help or advice. Others expressed a desire for ongoing help with their CVs, job searching, job applications, and preparing for any interviews, as well as transitioning into work. Some participants also wanted help accessing education or training courses that would improve their chances of gaining employment. For example, one participant said that she would like help starting a computer course, whilst another who had disengaged from IPS indicated that she wanted to reengage so that she could get support accessing a nursing course:

“I want to go back into nursing, but I do know that I've got to do like a six-month top-up course. So, to me, obviously once I've worked for, once I've got off the methadone, I would like to be, if I could get back in touch with IPS and get them to help me find out, you know, like filling the forms in, because I wouldn't have a clue.” (Participant 43, Female, Wave 3)

At their final interview (Wave 4), some participants commented that they did not feel they needed any further help from IPS as they had already secured their desired employment or felt confident that they could secure work independently. However, others continued to identify future support needs, including on-going help with writing CVs; searching or applying for jobs; careers advice; accessing training; managing welfare benefits; and confidence building. A few participants added that they would like their IPS workers to offer them the same type and level of support they were currently receiving for the foreseeable future.

#### 3.4.2 Potential improvements to IPS

When asked how IPS might be improved, only a few participants made suggestions at Wave 1. Their recommendations included having more frequent meetings with their employment specialists, holding meetings in a more private setting (such as an office rather than a coffee shop), and better links between IPS and substance use treatment services as the two could feel disconnected. At Wave 2, a small number of participants added that IPS was too narrowly focused on employment, and they would have liked more support enrolling on educational courses or training. Additionally, one participant thought that IPS should be promoted more widely as many people who might benefit from it do not know it exists. Meanwhile, another said that he would have liked more psychosocial support:

“I don't know if IPS also does a lot of other support beyond CV stuff and things like that. Which obviously if IPS does do that, then great. If not, I would say it would be good if they did do that. Not just like coaching for interviews, but I think other stuff like – I want to say therapy but maybe something to do with confidence and things like that as well.” (Participant 28, Male, Wave 2)

Participants similarly had few suggestions for improving IPS at Wave 3. Two thought that the program could be promoted better as they knew people who needed the support but were not aware of its existence; one male thought that it would be helpful if employment specialists had closer relationships with prospective employers; and another thought that there should be a designated office or space to have meetings as he frequently had to meet his employment specialist in locations that were inconvenient or unsuitable. Finally, one participant suggested that it would have been helpful to have been given written information after each meeting as he found it difficult to remember everything he had been told:

“Maybe a pamphlet or something, or something that gives me a record of all the information or something. Because I always tend to forget stuff sometimes. Maybe if I had a record of an appointment or what I should do, that might be good.” (Participant 11, Male, Wave 3)

At their last interview, many participants reported that IPS could not be any better. Countering this, however, several stated that there should be additional support for clients once they were in work. This included more opportunities to re-engage with IPS if clients decided to change jobs or careers, and more ‘check-ins’ during the first year of employment in case they had further questions or support needs:

“I think maybe another check-in would be good, maybe at six months and then maybe a year point would be good.” (Participant 48, Female, Wave 4)

Other suggested improvements were only made by one or two individuals but included providing more information about IPS prior to program enrolment; prioritising people who were further along in their recovery and had already established some stability; ensuring greater continuity when IPS workers changed; offering more support with managing welfare benefits; and giving greater priority to clients’ preferences when suggesting potential jobs:

“[Employment specialists should] not just search for anything to get somebody into work. Actually, take the time to ask this person if they, what they feel comfortable doing… So, that is the only one thing I'd say, to just make sure that the person is going to be happy in that job. Because there’s no point going to work and being unhappy because it doesn't work, does it?” (Participant 30, Male, Wave 4)

## 4 Discussion

Our analyses focused on clients’ views and experiences of IPS. Below, we summarize key findings by the relevant RE-AIM dimension, highlighting any changes over time. Given the dearth of prior research on people accessing IPS for alcohol and other drugs, we also draw comparisons with published literature on how people with severe mental illnesses (SMIs) view and experience the program. From this, we assess how IPS for people with substance use is currently working in England and whether any improvements can be made.

### 4.1 Reach

People in treatment for substance use problems who joined IPS tended to have limited knowledge and understanding of the program, particularly during the early stages of their engagement. Although IPS for substance use is only delivered to people receiving structured treatment, participants in our study did not always learn about the program through their treatment provider and were often uncertain who their employment specialist was. Over time, their knowledge of IPS increased but this was largely through their personal experiences of the program rather than because of any information provided to them. Despite this lack of knowledge, participants described good engagement with IPS. This level of engagement decreased at later study Waves but often because participants had secured jobs, were confident in job seeking themselves or had other life priorities (rather than because they disliked the program or found it unhelpful). Less positively, some who had disengaged wanted to re-join the program but did not understand how they could do this or had been told that they were no longer eligible.

The goal of achieving national coverage of IPS in substance use treatment services across England was close to being met at the time of our fieldwork (40). Moreover, IPS teams employed a range of strategies (including engaging with clients and promoting IPS to substance use workers) to raise awareness of the program (40). Latterly, race equity action plans designed to address barriers to access and other structural inequalities have also been developed (59). Within the published literature on IPS for people with SMIs, there is no evidence that clients lack awareness of the program or experience any difficulties accessing support from IPS once they have disengaged. Our study may thus have identified something specific about delivering the program to people with substance use problems or, more likely, we found a lack of knowledge of the program due to the comparative newness of IPS for substance use compared to IPS for people with SMIs. Either way, our findings point to the need for more information for clients (particularly around the time they join the program), and better communication on when and how clients can re-engage if they exit the program.

Consistent with our findings, good engagement with IPS for substance use has also been found in the literature on IPS for people with SMIs, with SMI clients typically reporting that they feel more motivated to work with their employment specialists than with other vocational professionals (47, 49, 51, 52). Studies on IPS for SMI have also found that clients sometimes disengaged from the program before securing work because they wanted to focus on their health or preferred to secure education or training or did not feel that IPS could help them with their job seeking any more (53, 60, 61). Nonetheless, there is evidence that employment specialists continue to support people with SMIs for longer than a year even when they have secured work, and that people with SMIs appreciate this assistance as it helps them to feel more settled in their jobs and able to change roles if they find that their current employment is unsuitable (62–64). These findings suggest there is scope for routinely extending the duration of support offered through IPS to people in treatment for substance use.

### 4.2 Effectiveness

From the perspectives of people in treatment for substance use, IPS provided multiple benefits. Very soon after joining the program, participants reported that they had already signed up to an employment agency or were applying for jobs or attending interviews. Over the course of the study, a notable proportion secured full- or part-time paid work which they said afforded them financial, psychological and social benefits. Others had moved closer to the labour market by accessing education, training or volunteering opportunities or by increasing their confidence, optimism or motivation for work. Even when participants had found a job independently of IPS they generally felt that the program had been instrumental in the process. Reflecting these outcomes, participants were very positive about IPS, using terms such as ‘great’, ‘amazing’ or ‘brilliant’. They also expressed confidence that their employment specialist would help them find a job and were appreciative of all the support they had received. Indeed, there were only a few criticisms of IPS, and these tended to arise because people wanted more, rather than less, assistance from program.

As our study was qualitative, we cannot draw any wider inferences about the success rate of IPS in terms of helping people into paid work. However, national data on clients receiving IPS for substance use in England indicate that around half obtain employment within 18 months of beginning the program (45). Clients of IPS for SMI have also reported positive outcomes in terms of securing paid/competitive full- or part-time work (50, 52, 53, 62–64), entering education, training or volunteering (49, 60–62, 64), and/or feeling more confident, positive or motivated for employment (46–48, 51, 52). Collectively, these findings suggest that the effectiveness of IPS should not only be measured in terms of how many people secure a competitive paid job. It is also important to assess wider outcomes, such as movement closer to the labour market through upskilling, applying for jobs, attending interviews, or simply feeling more interested in, and able to, work.

Paralleling our findings, clients of IPS for SMI were largely positive about the program and the support they received (46–49). Similarly, they expressed few criticisms and those criticisms that arose tended to relate to wanting more support rather than less support or a different kind of help. For example, some participants in a six-centre international randomized controlled trial of IPS for people with psychotic disorders reported that they had wanted more support from their IPS workers including more in-work support and more assistance with benefits (52). Meanwhile, several clients of IPS for SMI in Norway commented that they saw their employment specialists less often after securing a job and they would have preferred more regular contact with them during this period (53). These findings indicate that IPS is imperfect, but the benefits of the program outweigh the negatives from the perspective of both clients with SMIs and clients in treatment for substance use.

### 4.3 Implementation

IPS provided a very wide range of support to people with substance use. This included various forms of practical employment-related assistance, such as help with CVs, job searching and applications, approaching employers, preparing for interviews, and enrolling on courses or training. In addition, employment specialists sometimes accompanied participants to interviews or meetings; helped them to set up bank accounts; facilitated access to bus passes, work clothes, laptops, driving lessons, driver’s licences, work certificates or identification; and completed welfare benefit calculations to check they would be better off in work. Many participants also reported that their employment specialist had provided crucial emotional support which had enabled them to feel motivated to work and given them the confidence and self-belief they needed to feel that employment was viable for them. Accordingly, our analyses showed how participants valued both the practical support that IPS afforded them but also the open and trusting relationships and regular contact they had with their employment specialists.

These findings are consistent with the types of support documented in studies of IPS for SMI. Thus, people with SMIs have also reported that they received practical help with creating, editing and disseminating their CVs (46, 48, 62), job searching (including setting up online accounts and looking for jobs online and via social networks) (47, 48, 51, 53, 62), preparing for interviews (e.g., by doing mock interviews) (51), enrolling on educational or training courses (46, 50, 61, 63), and organizing finances (46, 47, 49, 50). The wide range of practical support received by participants in our study and other research indicate that IPS is being delivered as intended; that is, flexibly and in a person-centred way to meet each client’s specific employment-related needs.

The significance of the emotional support provided by employment specialists, particularly in building clients’ self-esteem and motivation for work, is also documented in the IPS and SMI literature. Clients of IPS for SMI have likewise emphasized how much they valued the relationships and regular contact they had with their employment specialists (47, 50, 51, 61), and they particularly appreciated it when workers took time to listen to them and encouraged them to establish their own aspirations and interests (47, 49–52). Indeed, having a poor relationship with an employment specialist (or another vocational professional) who was not encouraging left clients of IPS for SMI feeling demotivated (53, 60). Since the therapeutic relationship that employment specialists establish with their clients is so central to the overall experience of IPS, successful IPS implementation needs to invest in the knowledge, skills and personal attributes that IPS team members bring to their role. In England, however, short-term funding and the commissioning structure of IPS in substance use services mean that IPS staff work in small teams on fixed-term contracts which causes challenges for their recruitment and retention (40).

### 4.4 Maintenance

Despite their widespread appreciation of the program, some clients of IPS for substance use made suggestions for improvements. Recurrent ideas included better promotion of IPS, giving people more information about the program before they enroll, additional in-work support, increased help accessing education and training, more opportunities to re-engage if clients disengage or are discharged from the program, and more meetings with employment specialists in private settings. Occasional suggestions included more frequent meetings, additional face-to-face meetings, greater focus on clients’ personal job preferences, better links between IPS and substance use treatment services, extra psychosocial support, improved relationships between IPS and local employers, being given written information after meetings, prioritising people further along in their recovery, and greater continuity when employment specialists change. Looking to the future, many people were keen to have ongoing support from IPS, although this desire tapered off once people started working or felt more confident that they could secure work independently. Mostly participants wanted more of the same type of support, but also more in-work support (including someone who would stay in touch with them and on whom they could rely) and more help accessing education or training.

Studies of clients receiving IPS for SMI have not tended to present data on the types of assistance participants wanted from the service in the future. Nonetheless, consistent with our findings, research on IPS for SMI has indicated that clients appreciate the support they are already receiving and seem content to have either the same level of help (46, 51) or even more assistance going forwards (52, 53). Currently, clients of IPS for substance use in England receive support with soft time limits of 9 months for job-searching followed by 4 months of in-work support. There is, however, a further requirement that clients need to be receiving structured treatment for their alcohol or other drug use to be eligible. Since some people may desire ongoing support from IPS after their treatment for substance use has ended, it seems advisable to not only extend in-work support over a longer period but also to offer this to people who have exited treatment where clients want this.

It takes time to embed any new program into substance use treatment services, but our wider evaluation found that most IPS teams have integrated well within treatment services, and most IPS team members feel confident in their role (40). This does not mean that there will not be examples of poor practice and changes can always be made to ensure that the program is delivered more successfully over the longer-term. Indeed, some similar potential improvements to IPS for SMI have been reported (e.g., more in-work support, more help with benefits, more support securing education or training, and more attention to client preferences about jobs and when and where to meet) (52, 53, 60). Importantly, some of the improvements to IPS suggested in our study were only made by one or two individuals, some were consistent with how IPS should be delivered according to the 25-point fidelity scale, and some highlighted that participants only wanted more of what was already being offered. Overall, none of the suggested improvements seemed likely to threaten the future viability of the IPS for substance use program.

### 4.5 Limitations and strengths

As a qualitative study, our findings are limited by small sample size and the demographic characteristics of the participants interviewed (more males than females, mostly White British, and mostly people reporting alcohol as their main problem substance). Data from the first Wave of the study also showed that participants were often not long-term unemployed, so the sample may have been skewed towards those who were more ‘work ready’. We do not have information beyond 12 months to tell us how long people who secured paid employment stayed in their jobs. Equally, we do not know whether those who withdrew from the study had different and potentially less favourable views and experiences of the program than those who completed all four interviews. Despite this, we present original data on IPS for substance use and our methods have enabled us to explore changes over time and to capture the views and experiences of those who both secured and did not secure work. Having team members who had their own personal experiences of substance use and recovery was an additional strength that enabled us to build rapport with participants and helped to reduce unsupported academic assumptions when interpreting participants’ accounts. Finally, our full and open description of both our sample and data analysis processes, as well as clear consistencies with qualitative research on IPS for people with SMIs, indicate that our findings have credibility and transferability which are key components of trustworthiness in qualitative data analyses (65).

## 5 Conclusion

This paper has addressed a current gap in understanding by exploring clients’ views and experiences of IPS for substance use to understand how the program is working in England and whether any improvements can be made. Our analyses suggested that IPS could reach more of its target population if clients were provided with additional information and more support was routinely offered over a longer time frame, including to clients who exited substance use treatment. People with alcohol and other drug problems reported multiple benefits from IPS and were very positive about the program. In this regard, they valued increasing confidence and moving closer to the labour market as well as securing paid work. Evaluations of IPS should therefore include outcomes that capture clients’ subjective feelings, alongside objective measures of gaining competitive paid work. Consistent with the aims of IPS, practical employment-related support was provided in a flexible and person-centred way, but clients also valued the emotional support provided to them by their employment specialists. Investing in IPS staff (for example, through staff development and job security) therefore seems important to successful program delivery in the longer-term. Clients who suggested improvements to IPS often wanted more of the same type of support and were likely to disengage when their employment goals had been met. Together, these findings add to an emerging literature which shows that IPS can be delivered with promising results to people in treatment for substance use (21, 40, 42, 43).

## Data Availability

The datasets presented in this article are not readily available because of ethical and privacy considerations that could compromise participant anonymity. Requests to access the datasets should be directed to joanne.neale@kcl.ac.uk.
